# Esomeprazole’s Role in Enhancing Colonic Anastomotic Healing Post-Ischemic Injury in the Rat Model

**DOI:** 10.3390/medicina61050851

**Published:** 2025-05-06

**Authors:** Faruk Pehlivanli, Oktay Aydin, Mehmet Selçuk Mısırlıgil, Kevser Peker, İlker Kaplan

**Affiliations:** 1 Department of General Surgery, Kirikkale University School of Medicine, 71450 Kirikkale, Turkey; drfapeh@hotmail.com; 2 Department of General Surgery, University of Health Sciences, 06010 Ankara, Turkey; 3 Department of Anesthesiology and Reanimation, Kirikkale University School of Medicine, 71450 Kirikkale, Turkey; 4 Department of General Surgery, Ermenek State Hospital, 70400 Karaman, Turkey

**Keywords:** hypoxia reperfusion injury, colon, anastomosis, esomeprazole

## Abstract

*Background and Objectives*: Colonic anastomotic leaks are still a critical cause of morbidity and mortality. The study aimed to investigate the effects of esomeprazole on anastomotic healing after left colon anastomosis in rats with an ischemic colon. *Material and Methods:* Thirty-five male Wistar albino rats were divided into acute (CONTROL-A, ESP-A) and chronic (CONTROL-C, ESP-C) stage groups. Rats in the CONTROL-A and CONTROL-C groups underwent colonic anastomosis after hypoxia-reperfusion injury in the colon, and intraperitoneal saline was administered for three and ten days, respectively. Intraperitoneal 10 mg/day esomeprazole was given to the rats in the ESP-A and ESP-C groups for three and ten days after similar surgical procedures. Then, at scheduled times, 2 cm proximal and distal regions of the anastomosis line were resected, and bursting pressure was measured. Hydroxyproline (HYP), myeloperoxidase (MPO), malondialdehyde (MDA), caspase-3 (CSP3) and catalase (CAT), nitric oxide (NO), reduced glutathione (RGT), superoxide dismutase (SOD), TNF-α, IL-6, aspartate aminotransferase (AST), alanine aminotransferase (ALT) levels were measured in tissue and blood serum samples. *Results:* In the acute stage, CAT, NO, RGT, and SOD values in ESP-A group were lower than CONTROL-A group values. In addition, TNF, IL-6, ALT, and AST values in the ESP-A group were higher than the CONTROL-A group values between groups (*p* < 0.05). However, HYP and burst pressure values were not different between the groups. In the chronic stage, CAT, NO, RGT, SOD, CSP3, and burst pressure values in the ESP-A group were higher than CONTROL-A group values (*p* = 0.05). In contrast, TNF, IL-6, ALT, AST, HYP, MPO, and MDA values in the ESP-A group were lower than the CONTROL-A group values (*p* < 0.05). *Conclusions:* These results suggest that esomeprazole has anti-inflammatory and antioxidant activity in the chronic phase of ischemia–reperfusion injury, thus protecting the intestinal tissue from ischemic damage and enhancing the healing of the anastomosis line.

## 1. Introduction

In surgical practice, colorectal anastomoses are performed in many conditions, such as colon and rectum cancers, ulcerative colitis, Crohn’s disease, ischemic colitis, mechanical bowel obstruction, recurrent diverticulitis and trauma, and anastomotic leaks continue to be a significant complication affecting morbidity and mortality [1]. It harms the patient’s quality of life due to interventions such as relaparotomy and temporary or permanent stoma [2]. With the advances in laparoscopic colon surgery, laparoscopic colon anastomoses are performed more frequently today. Although the intraoperative and postoperative period is shorter, anastomotic leaks can be seen at a rate close to open surgery (0–20%), depending on the location of the colon segment where resection anastomosis is performed [3,4].

Ischemic colitis occurs when the blood flow in the colon is disrupted due to occlusive or non-occlusive causes of the mesenteric blood flow [5,6]. Ischemic changes that progress from the colonic mucosa to the serosa can produce transmural necrosis and cause gangrenous colitis requiring surgical intervention. If blood flow is restored after ischemic damage, it can also lead to reperfusion damage. This damage causes lipid peroxidation and cell necrosis in the intestinal cell membrane due to reactive oxygen species [7].

Proton pump inhibitors (PPIs) inhibit the hydrogen-potassium ATPase enzyme (HK-ATPase), consisting of a catalytic alpha and a regulatory beta subunit. HK-ATPase has two alpha subunits: the HKalpha-1 isoform found in kidney and gastric parietal cells, and the HKalpha-2 isoform found in colon cells [8]. Studies in the literature have suggested that esomeprazole may have healing effects on hepatic and lung fibrosis, cardiac stroke, and renal ischemia–reperfusion injury [9,10,11,12,13,14,15]. However, in the literature, no study has been found that demonstrates esomeprazole’s effects on colon ischemia–reperfusion injury.

This study aimed to explore the effects of esomeprazole on colon anastomosis healing in rats with mesenteric ischemia–reperfusion injury.

## 2. Materials and Methods

This study was conducted after receiving ethical committee approval from the Institute of Experimental Medicine and Research, decision number 39/dated 17 September 2018.

### 2.1. Animals

**Figure 1:**
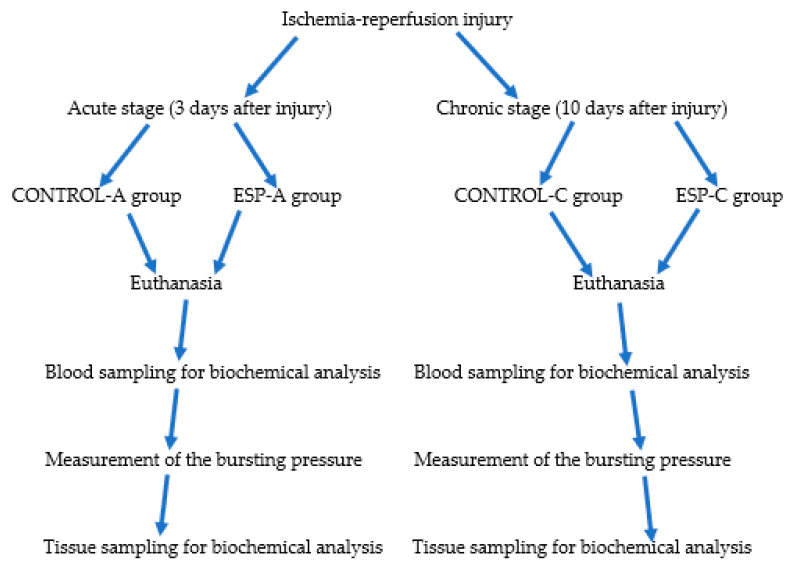
The figure shows the experimental workflow diagram and the distribution of experimental groups.

The experimental model included 40 10-week-old Wistar albino male rats weighing 300–350 g. To determine the study sample size, articles published in the literature that included a similar experimental model were used, and, therefore, no power analysis was applied. They were divided into acute (first three days after ischemia–reperfusion injury) and chronic stage (ten days after ischemia–reperfusion injury) groups below. Two control groups were formed to demonstrate the healing levels of the colonic anastomosis line in the acute and chronic phases of wound healing. Additionally, two more experimental groups were constructed to compare the therapeutic effects of esomeprazole in the acute and chronic stages of ischemic injury with the recovery rates in the control groups (Figure 1).

#### 2.1.1. Acute Period Groups

− CONTROL-A group (45 min of ischemia and then 45 min of reperfusion was performed over the colon mesentery, and then colon transection and end-to-end colon anastomosis was applied, and a single daily dose of 0.5 mL saline was administered intraperitoneally for three days, *n* = 10).
− ESP-A group (After 45 min of ischemia and then 45 min of reperfusion, the colon mesentery, colon transection, and end-to-end colon anastomosis were applied, and a single daily dose of 10 mg/day esomeprazole was administered intraperitoneally for three days, *n* = 10).

#### 2.1.2. Chronic Stage Groups

− CONTROL-C group (45 min of ischemia and then 45 min of reperfusion was performed over the colon mesentery, and then colon transection and end-to-end colon anastomosis were applied, and a single daily dose of 0.5 mL saline was administered intraperitoneally for ten days, *n* = 10).
− ESP-C group (45 min of ischemia and then 45 min of reperfusion were performed over the colon mesentery, and then colon transection and end-to-end colon anastomosis were applied, and a single daily dose of 10 mg/day esomeprazole was administered intraperitoneally for ten days, *n* = 10).

The dose of esomeprazole to be used in this study was determined both using the equation described by Nair et al. below and taking into account the doses used in some studies using esomeprazole in the literature [15,16,17]:

“Animal Equivalent Dose (mg/kg) = Human dose (mg/kg) × K_m_ ratio”

In this study, the intraperitoneal route was preferred for administering the experimental drug to the subjects. The literature reports indicate that intravenous drug administration is generally impractical for rodent studies and is not preferred, especially for chronic or recurrent treatments. Moreover, intraperitoneal administration is recognized as a valid parenteral route in the literature [18]. Therefore, the intraperitoneal route was chosen in this study due to its ease of administration, reproducibility, and good tolerance by the subjects. However, the pharmacokinetics of substances administered intraperitoneally are more similar to those observed following oral administration because the primary absorption route is the mesenteric veins, which empty into the portal vein before passing through the liver [18,19]. Consequently, in our study, a dose equivalent to the human oral dose of esomeprazole was administered intraperitoneally to the subjects to align with clinical applications, minimize pharmacokinetic risks, and achieve effective blood concentrations.

### 2.2. Surgery

**Figure 2:**
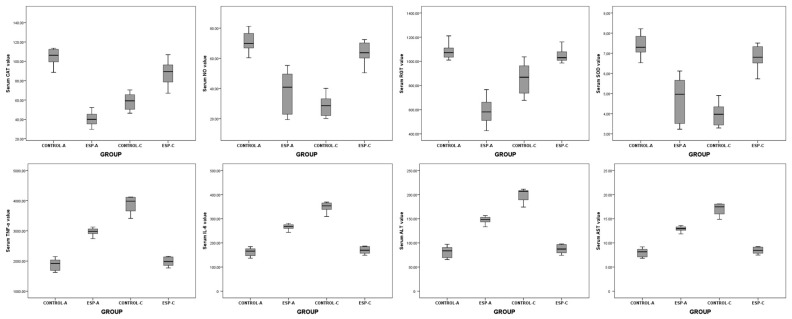
The graphs show the results of serum biochemical analysis of the acute and chronic period groups (CAT: catalase, NO: nitric oxide, RGT: reduced glutathione SOD: superoxide dismutase, TNF: tumor necrosis factor, IL6: interleukin 6, ALT: alanine aminotransferase, AST: aspartate aminotransferase).

All rats were given intramuscular sedation anesthesia with 50 mg/kg ketamine hydrochloride (Ketalar, Pfizer, New York, NY, USA) and 5 mg/kg xylazine (Rompun^®^ 2%; Bayer, Bayer, Germany). Then, using 10% povidone-iodine, antisepsis was applied to the area to be operated on in the supine position. Sterility conditions were ensured throughout the surgery, and spontaneous respiration of the rats was allowed. Following this, a 3 cm median incision was made to the abdomen, and the cecum and large intestines were externalized from the abdomen. The ascending colon mesentery, including its collaterals, was clamped with atraumatic “bulldog” clamps (VASCU^®^Stop, BMXS-006/BMXC-007, Penumbra, Inc, Alameda, CA, USA), and 45 min of ischemia was achieved in the colon. Then, the clamps were removed to provide reperfusion, and the tissues were allowed to reperfuse for 45 min. Following reperfusion, the ischemic descending colon was fully transected, and then a single-layer end-to-end (colon-to-colon) anastomosis was created using 8–10 interrupted and equidistant stitches spaced 4 mm apart using 6/0 polypropylene (Figure 2). After this procedure, the externalized intestinal segments were placed back into the intra-abdominal cavity, and the abdominal fascia, followed by the full-thickness skin of the rats, were closed continuously with 3/0 polypropylene sutures anatomically. Then, intraperitoneal 10 mg/day of esomeprazole was administered to rats in the ESP-A and ESP-C groups once daily. In addition, intraperitoneal 0.5 mL saline was administered to the rats in the CONTROL-A and CONTROL-C groups once daily.

On the third postoperative day, the CONTROL-A and ESP-A groups of rats, and on the tenth postoperative day, the CONTROL-C and ESP-C groups of rats, were re-anesthetized intramuscularly with 50 mg/kg ketamine hydrochloride and 5 mg/kg xylazine while in the supine position. Re-exploration was performed via midline incision, the anastomosis line was found, and the intestinal segment where the anastomosis line was located, together with two centimeters of proximal and distal regions, was exteriorized and resected. After the collection of tissue samples, the rats were euthanized by taking blood from the whole body by cardiac puncture, and the blood samples taken by puncture were centrifuged (Nüve NF1200, Nüve NF1200R, NÜVE, Saracalar, Akyurt, Türkiye) at 3000 rpm for ten minutes. Then, the obtained sera were stored at −80 °C.

### 2.3. Anastomotic Burst Pressure Measurement

**Figure 3:**
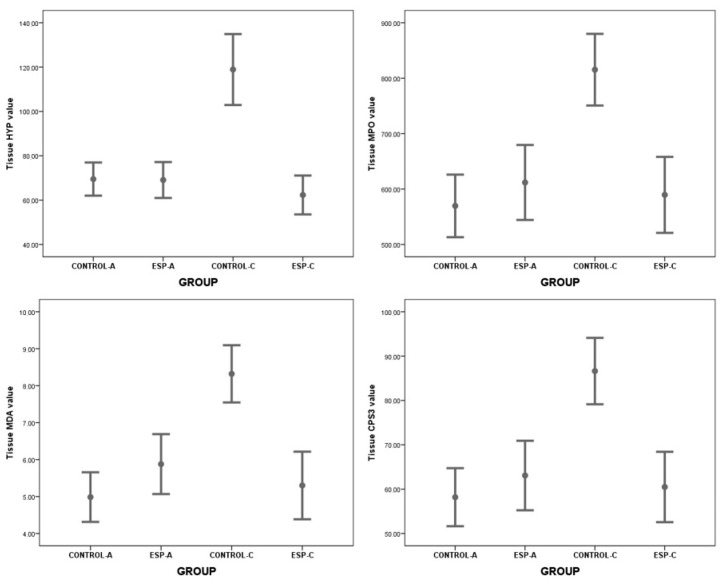
The graphs show the results of the biochemical analysis of colon tissues of the groups belonging to the acute and chronic periods (HYP: hydroxyproline, MPO: myeloperoxidase, MDA: malondialdehyde, CSP3: caspase 3).

The lumen of the resected intestinal segments of all rats was cleaned of fecal content with warm saline. Then, the distal part of the removed colon segment with anastomosis was tied tightly with 4/0 silk. The catheter of the infusion pump (Braun^®^ Infusomat Space, B. Braun, Melsungen, Germany) was inserted into the intestinal lumen from the proximal end of the intestinal segment. A manometer was connected to the infusion pump with a three-way cannula, and saline was infused through the catheter into the intestinal lumen through this catheter line at a constant rate. At the same time, the pressure was measured in the lumen. Bursting pressure was recorded as the highest value before evident saline leakage or sudden pressure loss was detected (Figure 3). After the burst pressures were recorded, the anastomotic line was stored at −80 °C for biochemical analyses.

### 2.4. Biochemical Analysis

Frozen tissue samples were homogenized with PBS (phosphate-buffered physiological serum, pH: 7.4) solution, and hydroxyproline (HYP) (µg/mL) (HYP, Catalog No: E-BC-K061-S, Elabscience, Houston, TX, USA), myeloperoxidase (MPO) (u/g) (MPO, Catalog No: RLM95, Rel Assay Diagnostics, Gaziantep, Turkey), malondialdehyde (MDA) (nmol/g) (MDA, Catalog No: RLMD0158, Rel Assay Diagnostics), and caspase-3 (Caspase-3, Catalog No: E-EL-R0160, Elabscience) values in the tissue samples were measured by the ELISA method in a microplate reader (Catalog No: 2011-06, Thermo Scientific Multiskan FC, Waltham, MA USA).

After thawing the frozen serum samples at room temperature, catalase (CAT) (kU/g Hb) (CAT, Catalog No: RLD8934, Rel Assay Diagnostics), nitric oxide (NO) (nmol/g) (NO, Catalog No: RLN864, Rel Assay Diagnostics), reduced glutathione (RGT) (mmol/g) (G-Reductase, Catalog No: E-EL-R1127, Elabscience), superoxide dismutase (SOD) (U/mL) (SOD, Catalog No: RLD0123, Rel Assay Diagnostics), tumor necrosis factor-alpha (TNFα) (pg/mL) (TNFA, Catalog No: E0764Ra, BT LAB, Saint Louis, MO, USA), interleukin-6 (IL-6) (pg/mL) (IL-6, Catalog No: E0135Ra, BT LAB), aspartate aminotransferase (AST) (u/L) (AST, Catalog No: RLA785, Rel Assay Diagnostics), and alanine aminotransferase (ALT) (u/L) (ALT, Catalog No: RLL865, Rel Assay Diagnostics) level values were measured by the ELISA method in a microplate reader.

### 2.5. Statistical Analysis

SPSS 21.0 (IBM, New York, NY, USA) package program was used for statistical evaluation. The *Shapiro–Wilk* test was used to test the normal distribution of all data. The values of the study parameters were recorded as mean, median, standard deviation, minimum, and maximum. An *Independent Samples t*-test was used to compare data showing normal distribution (*p* < 0.05). The *Mann–Whitney U* test was used to compare data not showing normal distribution (*p* < 0.05).

## 3. Results

During the study, two rats in the CONTROL-A and two in the ESP-A groups in the acute phase and one in the CONTROL-C group in the chronic phase were excluded from the study due to death. Therefore, the study was completed with thirty-five rats.

### 3.1. Acute Stage Results

**Figure 4:**
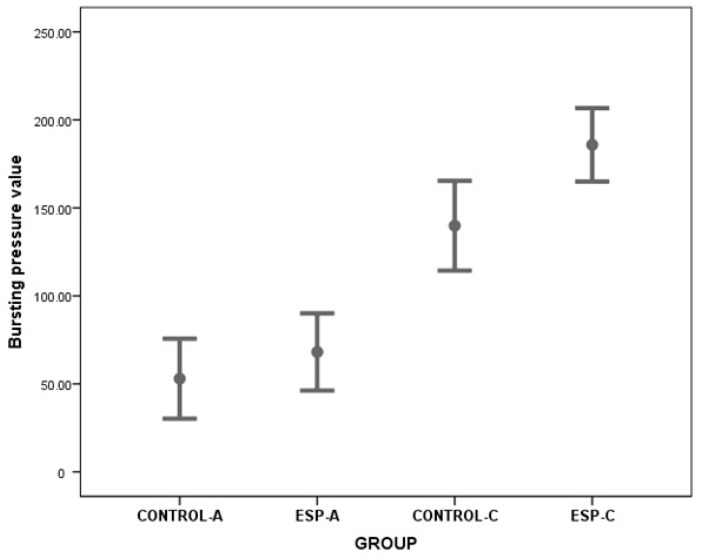
The graphs show the bursting pressure values of the colon anastomosis line of the groups belonging to the acute and chronic periods.

The serum CAT (Z = −3.361, *p* = 0.001), NO (*t* = 6.003, *p* < 0.001, 95% Confidence Interval (CI) 21.409–45.209), RGT (Z = −3.361, *p* = 0.001), SOD (*t* = 6.002, *p* < 0.001, 95% CI 1.729–3.651), TNF (*t* = 7.413, *p* < 0.001, 95% CI −1608.215–−886.433), IL-6 (*t* = 7.002, *p* < 0.001, 95% CI −158.100–−83.955), ALT (*t* = 7.413, *p* < 0.001, 95% CI −97.761–−53.884), AST (*t* = 7.404, *p* < 0.001, 95% CI −7.254–−3.995) values measured in serum samples were different between the groups. However, burst pressure values and tissue HYP, MPO, MDA, and CSP3 level values were not different between the groups (Table 1, Figure 2, Figure 3 and Figure 4).

These results showed that in the acute stage, CAT, NO, RGT, and SOD values in ESP-A group were lower than CONTROL-A group values. In addition, TNF, IL-6, ALT, and AST values in the ESP-A group were higher than in the CONTROL-A group. With these results, it could be said that although esomeprazole could have antioxidant properties in the serum samples, it could not have an anti-inflammatory effect in the tissue samples, and, therefore, it could not improve the burst pressure values and healing of the colon anastomosis in the early stage of the colon ischemia–reperfusion injury.

### 3.2. Chronic Stage Results

The serum CAT (*t* = −3.366, *p* < 0.001, 95% CI −40.289–−20.232), NO (Z = −3.674, *p* < 0.001), RGT (Z = −3.103, *p* = 0.002), SOD (Z = −3.674, *p* < 0.001), TNF (Z = −3.674, *p* < 0.001), IL-6 (Z = −3.674, *p* < 0.001), ALT (Z = −3.674, *p* < 0.001), AST (Z = −3.674, *p* < 0.001) values were different between the groups. The tissue HYP (*t* = 7.315, *p* < 0.001, 95% CI 40.257–72.893), MPO (*t* = 5.428, *p* < 0.001, 95% CI 138.007–313.520), MDA (*t* = 5.670, *p* < 0.001, 95% CI 1.896–4.144), and CSP3 (*t* = 5.428, *p* < 0.001, 95% CI 15.976–36.292) level values and bursting pressure values (*t* = −3.216, *p* = 0.005, 95% CI −76.243–−15.659) were different between the groups (Table 2, Figure 2, Figure 3 and Figure 4).

These results showed that serum CAT, NO, RGT, SOD, and tissue CSP3 level values, and burst pressure values in the ESP-A group were higher than in the CONTROL-A group values. In contrast, serum TNF, IL-6, ALT, AST level values, and tissue HYP, MPO, and MDA values in the ESP-A group were lower than in the CONTROL-A group. With these results, it could be said that esomeprazole could have antioxidant and anti-inflammatory properties in the serum and tissue samples, and it could improve the burst pressure values and healing of the colon anastomosis in the late stage of the colon ischemia–reperfusion injury.

## 4. Discussion

The most important factor responsible for the formation of ischemia–reperfusion injury (I/R) is thought to be superoxide anion (O^2−^) [20]. On the other hand, neutrophils reaching the damaged tissue after ischemia release many mediators such as myeloperoxidase (MPO), lysozyme, defensins, cathepsin G, elastase, protease 3, and azurocidin, and contribute to inflammation. In addition, matrix metalloproteinase (MMP) and lactoferrin mediators are released, and an antibacterial effect occurs, helping to remove dead tissues from the environment [21]. Superoxide anion is converted to less harmful hydrogen peroxide (H_2_O_2_) by the enzyme superoxide dismutase (SOD). However, while this transformation occurs, the complement system is activated with endothelial damage at the wound site and SOD is inhibited. Thus, the activated complement system causes the superoxide anion to remain high [22].

The radical formation further deepens the endothelial damage in a vicious cycle. The release of IL-1, PAF, PG I2, PG E2, growth factors, endothelin, NO, and thromboxane A2 (TxA2) increases as the endothelial damage increases. TxA2 increases the production of hydrogen peroxide from neutrophils. As a result, arterial vasoconstriction and venous vasodilation occur [23]. The complement system is also activated after endothelial damage. The complement system increases the levels of macrophage inflammatory protein-1a (MIP-1a), MIP-1b, MIP-2, monocyte chemoattractant protein (MCP)-1, TNF-α, IL-1, and IL-6, causing an increase in the inflammatory response. The activation of the complement system also leads to an increase in I/R damage by decreasing the levels of SOD alone during reperfusion [22]. In addition, nitric oxide is synthesized with the help of inducible nitric oxide synthase (iNOS) in the wound endothelium. To form peroxynitrite (ONOOH), NO combines with superoxide anion, which creates the most important effect in ischemia–reperfusion. Peroxynitrite can also transform into hydroxyl radical, which is known as the most harmful radical for the cell [24].

In addition, in the later period, angiogenesis, epithelial cell migration, and new capillary formation occur with the stimulation of tumor necrosis factor-α (TNF-α) and vascular endothelial growth factor (VEGF). Strengthening of epithelialization with epithelial cell migration acts as a barrier in the wound area, helps protect the tissue against infections, and prevents water loss. In addition, EGF released from platelets and macrophages is a critical factor for epithelial proliferation and chemotaxis [25].

When the findings of the present study were examined, it was observed that the CAT, NO, RGT, and SOD levels measured in the serum of the subjects in the ESP-A group in the acute period of ischemia–reperfusion injury were significantly lower than those in the CONTROL-A group. In contrast, the TNF, IL-6, ALT, and AST levels measured in the serum of the subjects in the ESP-A group were higher than those in the CONTROL-A group. With these results, it was thought that esomeprazole had antioxidant and anti-inflammatory activity in the acute period of ischemia–reperfusion injury, but those effects were weak in injured tissues. The fact that the serum TNF-α and IL-6 levels were measured higher in the ESP-A group compared to the CONTROL-A group values suggested that this agent could increase inflammatory processes through hematological pathways. Indeed, the increase in liver enzymes also supported this idea. With these findings, it was concluded that esomeprazole had antioxidant activity in the acute period, but its anti-inflammatory activity was weak, and it could not protect the liver from the secondary effects of colonic ischemia. On the other hand, since this study did not contain the results of histopathologic and immunohistochemical examination of the colonic anastomosis line, the inflammatory response and inflammatory cell infiltration levels in response to the acute stage of the ischemia–reperfusion injury and colonic anastomosis wound could not be shown in this study.

In the chronic period of ischemia–reperfusion injury, it was observed that the CAT, NO, RGT, and SOD levels measured in the sera of the subjects in the ESP-C group were higher than in the CONTROL-C group. Thus, it was thought that esomeprazole may have antioxidant activity in the chronic period. In addition, it was determined that the TNF, IL-6, ALT, and AST levels measured in the sera of the subjects in the ESP-C group were lower than in the CONTROL-C group. Thus, it was seen that esomeprazole could have anti-inflammatory and hepatoprotective activity. In addition, the MPO, MDA, and CSP3 levels measured in the tissues were significantly lower in the ESP-C group than in the CONTROL-C group. These findings suggested that esomeprazole showed an anti-inflammatory effect by reducing myeloperoxidase levels in ischemic intestinal tissue and an antioxidant effect by reducing malondialdehyde levels. It is also thought that it could suppress apoptosis processes by reducing caspase 3 levels and may have a healing effect on damaged cells undergoing apoptosis, thus having an anti-apoptotic effect. However, since the results of histopathologic and immunohistochemical examination of the colonic anastomosis line were not included in this study, the inflammatory response and changes in inflammatory cell infiltration levels in response to the chronic stage of ischemia–reperfusion injury and colonic anastomosis wound could not be demonstrated in this study. In addition, fibrosis levels in the anastomosis line could not be determined histopathologically due to this inadequacy. On the other hand, the literature reported that, to demonstrate therapeutic efficacy, esomeprazole must reach peak plasma levels (C-max), but the required dose-time curve is long [24]. It is therefore considered that the antioxidant, anti-inflammatory, antiapoptotic, and hepatoprotective effects of esomeprazole do not become apparent in the early stages of ischemia–reperfusion injury and become evident in the later stages, which may be related to this long dose-time curve.

The cells that play a major role in wound healing are fibroblasts and endothelial cells. Fibroblasts come from the surrounding tissues to the wound area, where permeability increases in the first twenty-four hours of the injury, and reach their highest level on the third to fifth day. Endothelial cells emerge from new capillaries formed by angiogenesis at the wound edge [26]. Fibroblasts provide collagen synthesis, especially collagen type 3, and when collagen, fibronectin, and all matrix elements reach sufficient levels, fibroblasts transform into myofibroblasts, and contraction is achieved [27]. Proline must be converted to hydroxyproline for collagen synthesis. During collagen synthesis, the most critical component of tissue tensile strength, the amino acids lysine and proline, must be hydroxylated [28].

The tensile strength of the maturing wound site is related to the number and shape of the cross-links in the collagen organization. The tensile strength of collagen reaches 30% after 3 weeks and 80% after 3 months. However, the tensile strength cannot increase by more than 80% of the pre-injury strength [27]. Tensile strength is provided primarily by the submucosa in the gastrointestinal tract. The submucosa consists of elastic fibers and 68% type I, 20% type III, and 12% type IV collagen. Tensile strength occurs earlier in wound healing in the intestinal wall than in other tissues [29].

In the present study, bursting pressure values of the anastomosis line were not different between the groups in the acute period of ischemia–reperfusion injury. In addition, hydroxyproline levels were similar between the groups in this period. It was thought that this was because there was not enough fibroblast migration in the anastomosis line in the acute period, and, therefore, sufficient collagen synthesis could not be developed. In addition, it was thought that the burst pressures were similar between the groups because there were not enough myofibroblasts in the environment. So, the wound contractile strength could not be provided sufficiently. However, when the numerical data were examined, it was still thought that the bursting pressure values of the ESP-A group were higher than CONTROL-A group. Therefore, esomeprazole could increase the contractile strength in the anastomosis line to some extent.

The literature reported that low hydroxyproline levels reflect a decrease in collagen synthesis, and, consequently, a decrease in tissue strength [30]. Conversely, the bursting pressure values in the ESP-C group were higher than in the CONTROL-C group during the chronic period of ischemia–reperfusion injury. This finding suggests that esomeprazole significantly increases the contractile strength of colonic anastomoses performed after ischemia–reperfusion injury. Therefore, it may have positive effects on anastomosis healing. However, tissue hydroxyproline levels in the ESP-C group were significantly lower than in the CONTROL-C group. Based on this finding, it was hypothesized that collagen synthesis in the anastomosis line decreased in the ESP-C group, leading to reduced tissue strength and resistance. However, the significantly higher anastomotic bursting pressure values in the ESP-C group did not support this theory, suggesting that this finding could be related to another important factor affecting anastomosis strength. Therefore, it was concluded that the histopathological changes at the ultrastructural level of this increase in tissue resistance due to esomeprazole warrant further analysis. Accordingly, advanced studies are needed to elucidate the pathophysiological events, such as histopathological examinations (including Masson–Trichrome and immunohistochemical staining) to determine collagen levels and fibroblast migration, as well as electron microscopy to examine collagen cross-linking.

This proton pump inhibitor drug is already used in clinical practice to reduce gastric acid secretion and prevent gastric complications, particularly in patients under stress. It is also known that the parenteral administration of this drug is the preferred method of administration, particularly for intensive care patients. To mirror clinical practice, esomeprazole doses equivalent to those administered to humans were given to the subject parenterally. Furthermore, the doses of the drug used in the study were almost identical to those used in clinical practice. Therefore, based on the clinical practice results of this drug and the results of this study, it was concluded that using this drug in patients who have undergone colonic anastomosis due to colon ischemia–reperfusion injury could greatly contribute to both preventing gastric complications and hepatic injury and promoting colon anastomosis healing. Therefore, it could be recommended that using this drug in such patients would be more appropriate.

### Limitations

This study had some limitations. First, a subject group that had not undergone ischemia–reperfusion injury was included in the study. Therefore, normal laboratory ranges of the study parameters could not be obtained. Second, due to the technical and financial restrictions, detailed histopathological and immunohistopathological examinations were not performed on the colon tissue and the anastomosis line. Finally, advanced biochemical examination methods (such as Western blot) that could reveal inflammatory processes in the tissues (such as ferroptosis and autophagy) could not be performed in this study because of the technical and financial restrictions. Although an advanced histopathological and biochemical examination could not be performed in this study, the biochemical data and burst pressure values showed that, besides its antiulcer activity, this pharmacological agent has anti-inflammatory and antioxidant activity and could positively affect colon anastomosis healing. Thus, it was concluded that this study is preliminary and can shed light on further studies.

## 5. Conclusions

Esomeprazole is currently the drug of choice for treating and preventing esophageal reflux, gastritis, and gastric ulcers in clinics and intensive care units. This study demonstrated that esomeprazole has anti-inflammatory and antioxidant effects in addition to its known anti-ulcer properties, and it could improve colonic anastomosis and repair ischemic colonic tissue and prevent the liver tissue from ischemic damage in rats undergoing colonic anastomosis after hypoxia-reperfusion injury. Thus, it is concluded that this drug can serve as a treatment option for patients with ischemic colon anastomosis in clinical practice, provided that the findings of this study are validated by advanced histopathological and biochemical analysis methods, dose optimization, and/or time course studies.

## Figures and Tables

**Table 1:** Comparative table of biochemical analysis results of acute stage groups.

		CONTROL-A	ESP-A			95% CI	
	Variable	Mean ± SD/ Median (min–max)	Mean ± SD/ Median (min–max)	*t*/*Z*	*p*	Lower	Upper
**Serum**	CAT	106.32 (88.61–148.63)	40 (29.65–52.31)	−3.361 †	**0.001**	-	-
	NO	71.01 ± 6.88	37.71 ± 14.11	6.003 *	**<0.001**	21.408	45.209
	RGT	1072.45 (1010.70–1211)	581.55 (426.30–766.20)	−3.361 †	**0.001**	-	-
	SOD	7.39 ± 0.56	4.70 ± 1.14	6.002 *	**<0.001**	1.727	3.651
	TNF	1810.97 ± 348.41	3058.29 ± 324.21	−7.413 *	**<0.001**	−1608.214	−886.432
	IL6	153.83 ± 37.23	274.85 ± 31.68	−7.002 *	**<0.001**	−158.100	−83.955
	ALT	76.87 ± 21.18	152.69 ± 19.71	−7.413 *	**<0.001**	−97.761	−53.884
	AST	7.67 ± 1.57	13.29 ± 1.47	−7.404 *	**<0.001**	−7.254	−3.996
**Tissue**	HYP	69.48 ± 8.95	69.08 ± 9.69	0.086 *	0.932	−9.600	10.405
	MPO	569.62 ± 67.56	611.85 ± 80.94	−1.133 *	0.276	−122.178	37.717
	MDA	4.99 ± 0.80	5.88 ± 0.97	−2.006 *	0.065	−1.847	0.062
	CPS3	58.19 ± 7.82	63.08 ± 9.37	−1.133 *	0.276	−14.142	4.365
	BP	53.00 ± 27.21	68.15 ± 26.23	−1.134 *	0.276	−43.810	13.508

(*) t value, Independent Samples t-test; (†) Z value, Mann–Whitney U test; p < 0.05. (SD: standard deviation, min: minimum, max: maximum, CAT: catalase, NO: nitric oxide, RGT: reduced glutathione SOD: superoxide dismutase, TNF: tumor necrosis factor, IL6: interleukin 6, ALT: alanine aminotransferase, AST: aspartate aminotransferase, HYP: hydroxyproline, MPO: myeloperoxidase, MDA: malondialdehyde, CSP3: caspase 3, BP: burst pressure).

**Table 2:** Comparative table of biochemical analysis values of chronic stage groups.

		CONTROL-C	ESP-C			95% CI	
	Variable	Mean ± SD/ Median (min–max)	Mean ± SD/ Median (min–max)	*t*/*Z*	*p*	Lower	Upper
Serum	CAT	59.00 ± 8.40	88.86 ± 11.81	−6.366 *	**<0.001**	−40.189	−20.232
	NO	28.65 (20.19–40.16)	63.69 (50.46–72.38)	−3.674 †	**<0.001**	-	-
	RGT	868.65 (677.40–1037.25)	1030.38 (986.25–1160.70)	−3.103 †	**0.002**	-	-
	SOD	3.97 (3.29–4.90)	6.81 (5.73–7351)	−3.674 †	**<0.001**	-	-
	TNF	3984.51 (3412.29–4960.28)	1983.39 (1769.88–2156.85)	−3.674 †	**<0.001**	-	-
	IL6	353.53 (309.45–460.73)	169.81 (148.94–186.75)	−3.674 †	**<0.001**	-	-
	ALT	206.73 (174.21–211.31)	87.35 (74.37–97.89)	−3.674 †	**<0.001**	-	-
	AST	17.47 (14.89–21.87)	8.45 (7.48–9.23)	−3.674 †	**<0.001**	-	-
Tissue	HYP	118.90 ± 20.81	62.32 ± 12.25	7.315 *	**<0.001**	40.257	72.893
	MPO	815.29 ± 84.12	589.53 ± 95.86	5.428 *	**<0.001**	138.007	313.521
	MDA	8.32 ± 1.01	5.30 ± 1.28	5.670 *	**<0.001**	1.896	4.144
	CPS3	86.62 ± 9.74	60.49 ± 11.10	5.428 *	**<0.001**	15.976	36.293
	BP	139.89 ± 33.19	185.84 ± 27.14	−3.216 *	**0.005**	−76.340	−15.659

(*) t value, Independent Samples t-test; (†) Z value, Mann–Whitney U test; p < 0.05. (SD: standard deviation, min: minimum, max: maximum, CAT: catalase, NO: nitric oxide, RGT: reduced glutathione SOD: superoxide dismutase, TNF: tumor necrosis factor, IL6: interleukin 6, ALT: alanine aminotransferase, AST: aspartate aminotransferase, HYP: hydroxyproline, MPO: myeloperoxidase, MDA: malondialdehyde, CSP3: caspase 3, BP: burst pressure).

## Data Availability

The original contributions presented in this study are included in the article. Further inquiries can be directed to the corresponding author.
